# Imported Loiasis at a Clinical Reference Center in Germany: A Retrospective Case Series

**DOI:** 10.4269/ajtmh.24-0022

**Published:** 2024-07-09

**Authors:** Tamara Nordmann, Julia Ruge, Dennis Tappe, Michael Ramharter

**Affiliations:** ^1^ Department of Tropical Medicine, Bernhard-Nocht-Institute for Tropical Medicine and I Department of Medicine, University Medical Center Hamburg-Eppendorf, Hamburg, Germany;; ^2^ German Center for Infection Research, Partner Site Hamburg–Lübeck–Borstel–Riems, Germany;; ^3^ National Reference Center for Tropical Pathogens, Bernhard-Nocht-Institute for Tropical Medicine, Hamburg, Germany;; ^4^ Centre de Recherches Médicale de Lambaréné, Lambaréné, Gabon

## Abstract

Loiasis is a rarely imported infectious disease that is often difficult to diagnose and treat. Here we describe clinical features and treatment outcomes of 11 patients with imported loiasis seen at a German reference center between 2013 and 2023. Clinical presentations varied by patient origin, with eye-worm migration and ophthalmological symptoms being more common among patients from endemic areas and Calabar swelling, subcutaneous swelling, and pruritus more prevalent among returning travelers from nonendemic regions. Eosinophil counts were higher in returning travelers. Diethylcarbamazine was most commonly used for treatment either as monotherapy in combination with ivermectin or with albendazole and ivermectin, respectively. In one patient, long-term follow-up indicated treatment failure after the first course of treatment. Another traveler was prescribed chemoprophylaxis with diethylcarbamazine after experiencing repeated infections due to long-term residence in a high-risk region in Cameroon.

More than 20 million people are estimated to be chronically infected with *Loa loa* and approximately 42 million people reside in regions of high or intermediate transmission.^1^ Because the most affected populations live in remote areas of the African rainforest with limited healthcare, clinical and laboratory research on loiasis is scarce.^2^ Published data predominantly stem from imported cases diagnosed and managed in countries outside the endemic regions in Italy, France, the United Kingdom, and the United States.^3^^–^^8^ Noteworthy differences in clinical manifestations have been described for individuals from endemic versus nonendemic countries, underscoring the importance of clinicians recognizing these differences when assessing suspected loiasis cases.^9^ In this case series, we describe imported cases of loiasis presenting at the Center for Tropical Medicine in Hamburg, Germany, between 2013 and 2023.

Data were retrospectively retrieved from the institutional electronic patient database. Loiasis episodes were defined according to the following criteria: 1) parasitological confirmation by microfilarial detection in the blood or 2) clinical diagnosis if eye-worm migration was confirmed (e.g., photo documentation, reports of ophthalmologist), Calabar swelling (localized, transient subcutaneous swelling, usually on the joints of the periphery) (Figure 1), or other symptoms suggestive for loiasis after exposure in an endemic region (e.g., pruritus, subcutaneous swelling, observation of worm-like structure under the skin) and laboratory markers suggestive for loiasis including eosinophilia (normal: absolute eosinophil count <0.5/mm^3^ and relative eosinophil count <5%), elevated IgE levels (normal: <158 kU/L), or positive filaria serology (in-house panfilarial IgG-detecting ELISA using *Dirofilaria immitis* extract as antigen).

**Figure 1.**
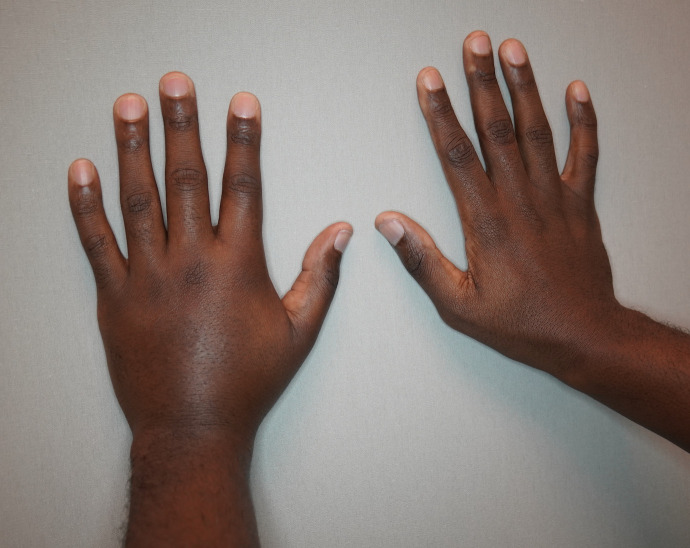
Transient Calabar swelling of the left hand observed in a patient with imported loiasis (Source: M. Ramharter).

Patients were from endemic regions with previous long-term exposure or returning travelers from nonendemic countries infected during a stay in a transmission region in Africa.

A total of 11 patients with *L. loa* infection (Table 1) were diagnosed and treated at a clinical reference center in Germany. Remarkably, one patient experienced three separate episodes of loiasis after repeated long-term exposure in a highly endemic area in Cameroon, with each occurrence successfully treated as evidenced by remission of clinical symptoms and normalization of eosinophile counts and IgE.

**Table 1:** Characteristics of loiasis patients stratified by patients from endemic country and returning travelers

	Patients from Endemic Countries (*N* = 7)							Median (IQR) or *N* (%)	Returning Traveler (*N* = 4)					RT with Three ep., 2nd ep.	RT with Three ep., 3rd ep.
									RT with One ep. (*N* = 3)			RT with Three ep., 1st ep. (*N* = 1)	Median (IQR) or *N* (%)		
Age at First Presentation	48	27	36	32	26	32	41	32 (27–41)	49	58	37	48	49 (43–54)	50	56
Sex	M	F	F	F	F	M	M		M	M	F	M		M	M
Exposure															
Country of Acquisition	Cam	Cam	Cam	Nig	Cam	CAR	Gabon		EG	Cam	Cam	Cam		Cam	Cam
Duration of Potential Exposure (Months)	?	300	420	264	228	96	492	282 (228–420)	1	1	1	8	1 (1–5)	3	29
Duration between Last Potential Exposure and Presentation at Clinic (Months)	14	36	4	132	83	0	0	14 (0–83)	2	5	2	3	2 (2–4)	1	1
Maximum Incubation Period (Months)	?	336	424	396	311	96	492	366 (311–424)	3	6	3	11	5 (3–9)	3	30
Clinical Signs and Symptoms															
Eye Worm	+	–	+	+	+	+	–	5 (71)	+	+	–	–	2 (50)	–	–
Calabar Swelling	–	–	+	–	–	+	+	3 (43)	+	–	–	+	2 (50)	+	+
Other Ophthalmological Symptoms	+	+	–	–	+	–	–	3 (43)	–	–	–	+	1 (25)	–	+
Subcutaneous Edema	–	–	+	+	–	–	–	2 (29)	+	–	+	–	2 (50)	–	+
Pruritus	+	+	–	–	–	–	–	2 (29)	+	–	+	–	2 (50)	–	–
Migrating Worm Under Skin	–	–	–	+	–	–	+	2 (29)	–	–	–	–	0 (0)	–	–
Headache	–	–	–	–	–	–	–	1 (14)	–	–	–	–	0 (0)	–	–
Abdominal Pain	–	–	–	–	–	–	–	0 (0)	–	–	+	–	1 (25)	–	–
Fever/Subfebrile Temperature	–	–	–	–	–	–	–	0 (0)	–	–	+	–	1 (25)	–	–
Laboratory Results															
Eosinophilia*	+	+	+	–	+	+	+	6 (86)	+	+	+	+	4 (100)	+	+
Mean Eosinophil Count (absolute/mm ^3^^)^	1.21	0.57	0.97	0.08	0.29	3.08	0.43	0.57 (0.29–1.21)	13.03	5.08	3.50	2.47	4.29 (2.99–9.06)	2.50	2.56
Mean Eosinophil Count (relative %)	31	11	14	2	6	39	6	11 (6–31)	55	40	40	20	40 (30–48)	20	21
Mean IgE Level (kU/L)	546	52	2,360	?	110	2,110	49	328 (52–2,110)	175	?	?	463	319 (n/a)	680	?
Microfilaremia	–	+	+	+	–	–	–	3 (43)	–	–	–	+	1 (25)	+	+
Mean Microfilarial Load (mf/mL), Range	Neg	1,667	3	5	Neg	Neg	Neg	5 (n/a)	Neg	Neg	Neg	70	70 (n/a)	600	12
Serology (*D. immitis* ELISA)	+	?	–	+	?	?	+	3 (43)	+	+	+	+	4 (100)	?	+
Treatment	IVM 200 µg/kg, single-shot (1 d) + DEC (21 d)	DEC (21 d)	IVM 200 µg/kg, single-shot (2 d) + DEC (21 d) + Albendazole ?	DEC (21 d)	NT	DEC (21 d)	NT		DEC (21 d)	DEC (?)	DEC (21 d)	DEC (23 d)		DEC (21 d)	DEC (21 d)
Adverse Events	–	–	Itch	Naus, emes, fatig, HTN	–	–	–		Arth	Itch, arth	Itch, dizz, fatig, HA	–		HA, itch, achill	Itch, dizz, fatig, HA
Treatment Outcome	LTF	LTF	Failure	LTF	LTF	Cure	LTF		Cure	LTF	LTF	Cure		Cure	LTF

? = unknown; achill = achillodynial; arth = arthralgia; Cam = Cameroon; CAR = Central African Republic; d = day; DEC = diethylcarbamazine; dizz = dizziness; EG = Equatorial Guinea; emes = emesis; ep. = episode; F = female; fatig = fatigue; HA = headache; HTN = hypotension; IQR = interquartile range; IVM = ivermectin; LTF = lost to follow-up; M = male; n/a = not applicable; Naus = nausea; Neg = negative; Nig = Nigeria; NT = no treatment; RT = returning traveler.

* Eosinophilia is defined as absolute eosinophil counts >0.5/mm^3^ or relative eosinophilia of >5%.

The majority of patients were from endemic regions in Africa (*n* = 7) and only four patients were travelers from nonendemic countries. Cameroon was the most common country of infection acquisition (*n* = 7; 64%) (Figure 2).

**Figure 2.**
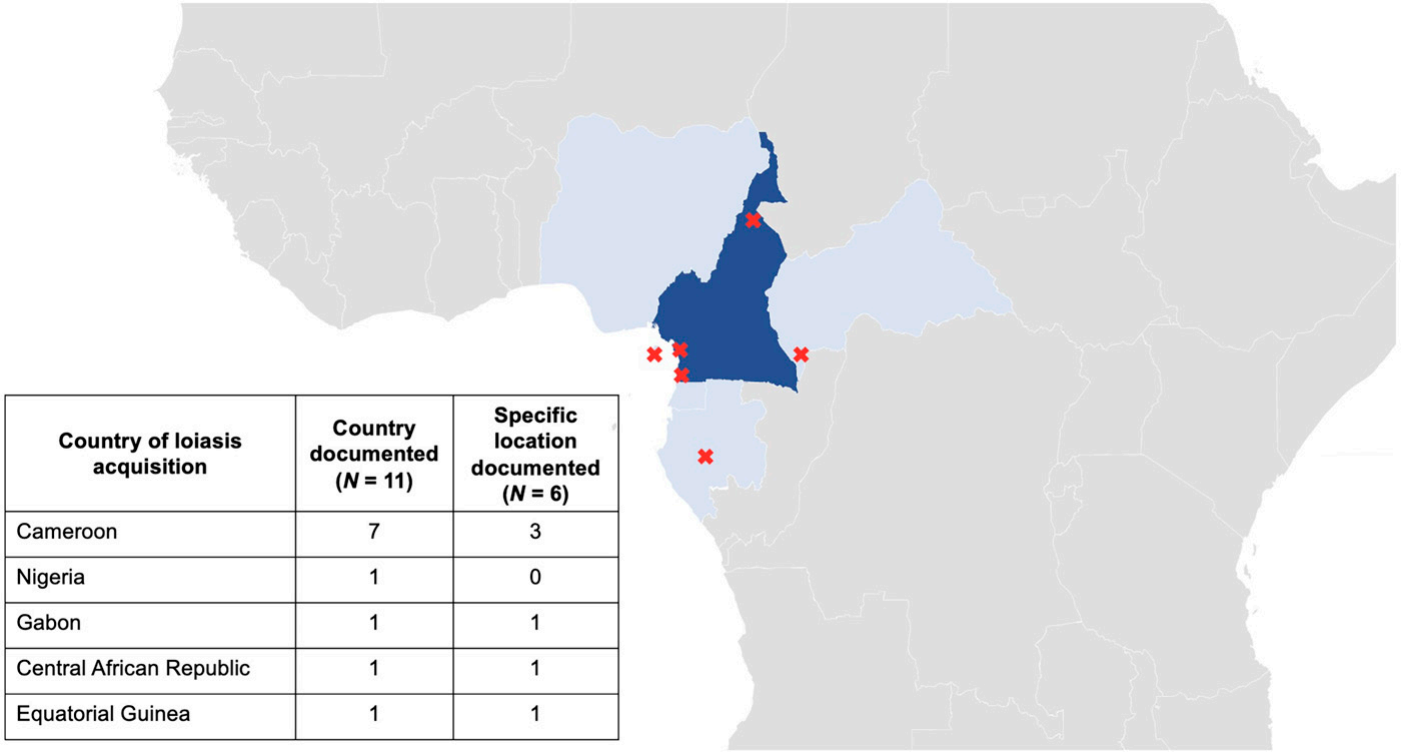
Geographic origin of imported loiasis cases. The country of acquisition was documented for all patients (*N* = 11), red cross: documented specific location of acquisition (*N* = 6).

Returning travelers from nonendemic regions were older (median age 49 years, interquartile range [IQR]: 43–54 years) and predominantly male (75%) compared with the patients from endemic countries with a median age of 32 years (IQR: 27–41 years) and 43% males. Returning travelers had a median of potential exposure of 1 month (IQR: 1–5 months) as cumulative time spend in an endemic area. Most patients from endemic countries had lived there for many years, resulting in a significantly extended potential exposure duration, with a median of 282 months (IQR: 228–420 months). In addition, median presentation to the healthcare system was 14 months (IQR: 0–83 months) after leaving the endemic area for patients from endemic countries, whereas returning travelers presented on within 2 months (IQR: 2–4 months) after their return to Germany.

All episodes of loiasis were symptomatic, with the pathognomonic signs being the most common. Eye-worm migration (71%) and other ophthalmological symptoms (43%) were more prevalent among patients from endemic areas, and 43% of *L. loa* patients from endemic countries initially consulted an ophthalmologist. In contrast, patients from nonendemic areas more frequently presented with Calabar swellings (50%), subcutaneous swelling (50%), and pruritus (50%) (Table 1). Only one episode each was associated with headache, abdominal pain, and subfebrile temperature, respectively.

Microfilaremia was confirmed in three loiasis patients from endemic countries by microscopic detection of microfilariae in the blood with a median microfilarial density of 5 mf/mL (range 3–1,667 mf/mL). Only one nonendemic traveler had detectable microfilaremia of 70 mf/mL.

Eosinophilia was reported for almost all loiasis episodes (91%). Travelers from nonendemic countries presented with higher median absolute eosinophil counts (4.29/mm^3^, IQR: 2.99–9.06 mm^3^) and median relative eosinophil counts (40%, IQR: 30–48%) than patients from endemic countries (0.57/mm^3^, IQR: 0.29–1.21 and 11%, IQR: 6–31), whereas median IgE levels were similar in individuals from endemic countries (328 kU/L, IQR: 52–2,110) and in individuals from nonendemic countries (319 kU/L).

Nine patients with loiasis were treated in an inpatient setting at our center, and two patients did not return for treatment initiation after establishing the diagnosis. Among the seven patients who received diethylcarbamazine (DEC) monotherapy, five reported mild to moderate post-treatment reactions, including itching, headache, emesis, nausea, joint pain, fatigue, and hypertension. Long-term follow-up data were available for three patients treated with DEC indicating treatment success.

To reduce microfilarial load before DEC treatment, ivermectin was administered in one patient from an endemic country without documentation of any adverse events. One patient initially rejected hospital admission for treatment, in whom a treatment cycle with albendazole was initiated. Subsequently, the patient agreed to be admitted for treatment with ivermectin and DEC. However, *L. loa* persisted after treatment, and an additional treatment cycle was recommended, which the patient declined.

One traveler from a nonendemic country who is a long-term resident in a high-risk area in rural Cameroon has been diagnosed repeatedly with loiasis. In the period between 2015 and 2023, he presented with three episodes of loiasis. Each episode was confirmed by the detection of microfilaria in the peripheral blood (median microfilaremia 70 mf/mL). In two episodes, he complained about impaired vision but never reported eye-worm passage. Each episode was treated with DEC, and he reported transient headache, itching, dizziness, fatigue, and achillodynia after treatment. No microfilaria were detected over a follow-up period of 6 months after the first two treatment cycles with simultaneous reduction of eosinophilia and IgE; therefore, he was declared cured after each treatment. However, when the patient returned to Cameroon after his third episode of loiasis, DEC 300 mg once weekly was prescribed for prevention of further infections.

This case series includes 11 imported loiasis cases treated at a German reference center. The main country of acquisition was Cameroon, aligning with findings in studies from France.^3^^,^^4^^,^^10^ Interestingly, retrospective analyses from the United Kingdom highlighted Nigeria as the primary source of imported cases,^5^^,^^7^ whereas in Spain Equatorial Guinea^8^ and in Italy the Democratic Republic of Congo were cited as the main origin of infection.^11^ These differences likely reflect distinct travel and migration patterns among European countries, explaining the variations in imported loiasis cases across Europe.

The diagnosis of loiasis is complex because a significant number of patients are amicrofilaremic. In these individuals, diagnosis relies mainly on pathognomonic symptoms including eye-worm passage or transient Calabar swelling.^1^^,^^12^ Additionally, laboratory markers such as eosinophilia, elevated IgE levels, or positive serology for filarial species are helpful but often nonspecific, particularly in patients from endemic regions.^13^

Consistent with previous findings on imported loiasis, patients from endemic regions more often presented with eye-related symptoms, including eye-worm migration, but with fewer overall symptoms compared with travelers from nonendemic countries. Almost all patients from endemic countries had eosinophilia, but mean eosinophil counts were lower than in travelers from nonendemic countries. Additionally, microfilaremia was detected more often.^3^^,^^5^^,^^7^^,^^9^^,^^11^^,^^14^ These consistent observations across imported loiasis cases suggest different immune responses to loiasis among different patient groups. Churchill et al.^5^ hypothesized that prolonged exposure reduces the immune response to the filarial infection over time, resulting in fewer symptoms and lower eosinophilia but a higher tolerance of microfilaremia.^1^^,^^15^ However, the exact immune mechanisms and potential influence of genetic factors of the parasite or the host are hitherto unknown.^9^^,^^16^

The differences in clinical and laboratory presentation are of importance for the diagnosis and management of loiasis.^17^^,^^18^ Mild symptoms and the absence of eosinophilia may lead to a delay in establishing the correct diagnosis and higher mean microfilaremia is associated with an increased risk for treatment related complications.^19^^,^^20^

Post-treatment adverse events were similar between patients from endemic and nonendemic countries in our series, all of which were reported as mild to moderate. No loiasis patient exhibited microfilarial loads, which would have constituted a contraindication for DEC treatment. Limited follow-up data hindered the systematic assessment of treatment effectiveness, however, which is a main limitation of this report.

Loiasis remains a highly neglected tropical disease. Physicians in nonendemic regions should consider loiasis as a potential diagnosis in patients exhibiting a relevant travel history to remote regions in central and western Africa and presenting with characteristic clinical or laboratory signs and symptoms. The consistently described differences in the clinical presentation of loiasis between patients from endemic regions and travelers from nonendemic countries highlight the need for further research to gain a better understanding of the pathophysiology of loiasis and its appropriate diagnosis and management.
